# Community Involvement: From Presentations to Participation

**DOI:** 10.1002/alz.087580

**Published:** 2025-01-09

**Authors:** Tara Tang, Athena Lavoie, Gregory A Brunson

**Affiliations:** ^1^ Butler Hospital, Providence, RI USA

## Abstract

**Background:**

Building a foundation of trust is the first step to establishing a relationship with the community that transcends any enrollment period. The Memory and Aging Program (MAP) Outreach Team formed in 2016 and has grown to a core of four team members in 2022, all focused on educating the public about Alzheimer’s Disease and Related Disorders (ADRD) and sharing opportunities to participate in research. This team explores different approaches to improve the access underrepresented populations (URPs) have to research and share some of their takeaways from community events.

**Method:**

The team specializes in building trust and two‐way communication with community partners. In 2023 MAP held ∼ 200 outreach events and presentations, 115 in underrepresented communities, where individuals were invited to join our prevention registry, and offered opportunities to learn about pathways to research participation. Health fairs held at community centers focused on the overall health of the BIPOC community and partnered with other organizations for health screenings, food and music. ApoE “swab parties” provided education and a low burden pathway to participate in research at cultural venues, alumni events and conferences. They offered time to meet MAP people in a warm environment to facilitate genuine conversations.

**Result:**

In combination with events and social media campaigns, the prevention registry has grown to over 5,300 enrollees, 16% of which are representative of URPs. Our swab parties generated 335 biospecimens, 7% enrolled in studies. The 2023 health fairs were attended by approximately 230 individuals with approximately 11% enrolling in our prevention registry. We established 20 new community connections and saw a 76% increase in URP events since 2022.

**Conclusion:**

It’s imperative researchers continue to refine best practices in diversity recruitment and enrollment to foster trust between URPs and investigators. Authentic connections are best made by hosting frequent events that focus on the community’s overall health and located in settings where people feel comfortable. We’re optimistic in 2024 with plans to translate our registry into Spanish, acquire a sprinter van to bring memory screens and blood draws to community events, and offer more studies in Spanish with an increase in bilingual staff.

